# ALST-W integrated index for enhanced surface temperature mapping of water bodies and vegetation using Landsat 8/9 satellite bands

**DOI:** 10.1038/s41598-025-09247-w

**Published:** 2025-07-09

**Authors:** Sam Navin MohanRajan, Agilandeeswari Loganthan, Prabukumar Manoharan

**Affiliations:** 1 School of Computing, Vel Tech Rangarajan Dr. Sagunthala R&D Institute of Science and Technology, Chennai, TN India; 2 School of Computer Science Engineering and Information Systems, Vellore Institute of Technology, Vellore, TN India

**Keywords:** Land surface temperature, Remote sensing, Geographic information systems, Land use/land cover, Land surface temperature, Adaptive land surface temperature of water bodies, Google earth engine, Environmental impact, Energy and society

## Abstract

Researchers are developing new methods to analyze changes in satellite data across various locations using remote sensing and geographic information systems (GIS). Land Surface Temperature (LST) maps are important indices for understanding changes in global land use and land cover (LU/LC). This study introduces the ALST-W (Adaptive Land Surface Temperature of Water Bodies) index to investigate the impact of water bodies on the LST map of the non-forest-covered Javadi Hills region, India, using Landsat 9/8 images for 2020, 2022, and 2024. The ALST-W results were compared with reference maps from Google Earth Engine (GEE), and the findings showed a good average accuracy of 95.06%. This study introduces the new index of the ALST-W, which displays the temperature data for high and low vegetation, along with the water bodies in a single raster map. The information from this work helps communities and policymakers understand environmental changes and take informed actions to protect vegetation and water bodies from significant future loss.

## Introduction

Ecosystems depend on a balanced mixture of natural components. It serves as the physical foundation for buildings and societies, and water provides essential support for everything from human consumption to agriculture and industry. Additionally, vegetation acts as a critical preservative in this framework. It helps to regulate the climate by cooling the air through shade and moisture release, sequesters carbon to counter global warming, and boosts biodiversity by providing homes for numerous species. This stabilizing effect spreads beyond the limits to areas near the forest zones, where vegetation acts as a defensive shield, maintaining natural health and connecting the fragmented natural habitats^1,2^. Research on LST-based vegetation and water bodies provides their role in justifying the heat stress in non-forest and forest-covered regions. Rising LST due to climate change and development can change the ecosystem balance, making it critical to investigate these dynamics. Water bodies control the land by acting as natural heat sinks, absorbing and dispersing heat through evaporation. They help reduce the city’s heat effect by lowering the temperatures in surrounding areas. Vegetation around the water bodies further enhances the cooling effect by providing shade and increasing moisture. Conserving and integrating the water bodies into landscapes is essential for regulating the LST and mitigating climate impacts^3,4^. The LST mapping has been considered active research for environmental monitoring. The landscape characteristics of a specific area and their impacts were analyzed using satellite images and geographic information technologies. With the help of satellite technologies, the LST change is analyzed and produced through different mathematical and algorithmic methodologies. Satellite images and spatial analytics help analyze past, present, and future patterns, supporting planners and agencies in sustainable resource management^5–9^. Satellites are equipped with remote sensing capabilities to provide varied spatial, spectral, and temporal data, which enables accurate monitoring of LST changes over time. Researchers used preprocessing techniques to minimize the atmospheric, radiometric, geometric, and topographic misrepresentations in raw satellite images^10–15^. The impact of LST changes through different spatial variables are slope, terrain shading, elevation, population data, rainfall data, Vegetation Index (VI), Bareness Index (BI), Snow Index (SI), Water Index (WI), and Built-up Index (BUI). A correlation analysis investigates the relationships and strength of connections among these land cover-based spatial factors^16–20^. LST impacts have been monitored over several decades worldwide, revealing temperature variations associated with vegetation and infertile lands. The effects of humans and other living things in nature will also affect the LST in many applications. The LST has continuously analyzed the deforestation rate, the development of aquatic ecosystems, burned area mapping, geothermal anomalies, agriculture monitoring, and volcanic activities. The Thermal infrared sensor (TIRS) bands were employed and processed to find the LST map. With a detailed analysis of LST from a research perspective, the influence of climate, heat waves, disasters, storms, floods, and droughts significantly outlines the surface temperature formation^21–25^.

Multispectral and hyperspectral imagery are both valuable tools for LU/LC classification and change detection in GIS^26–28^. Hyperspectral imagery provides high spectral resolution, allowing for detailed discrimination of land cover types and the detection of subtle environmental changes^29–34^. However, multispectral imagery is often preferred due to its greater availability, cost-effectiveness, frequent revisit cycles, and simpler processing requirements. It offers sufficient spectral detail for most LU/LC applications, making it a practical and efficient option for large-scale land monitoring and analysis^35,36^. Researchers worldwide have carefully explored the LST and its effects on LU/LC by presenting the key insights discussed in the following review. The Javadi Hills in Tamil Nadu, India, are home to diverse flora, including tropical dry deciduous forests with various medicinal plants and fauna comprising leopards and different bird species. This biodiversity supports ecological studies, particularly the LST changes, where non-forest areas like bushes and barren lands help highlight the vegetation and water-cooling effects for accurate LST mapping. The LST, VI, and BI information was analyzed using TIRS satellite data to measure the high and low vegetation cover across the forested and non-forested regions of Javadi Hills. The analysis showed that densely vegetated forest areas exhibited lower temperatures than warmer non-forested zones of Javadi Hills, India^13,37,38^. Water bodies and vegetation play a dynamic part in finding the LST in the Atai-Bhairab-Rupsha River confluence. The research displays a notable drop in maximum LST with increased vegetation (VI), consistent water body coverage (WI), and decreased barren land (BI)^39^. Rising LST on agriculture in Rajshahi was explored by examining the seasonal changes. Applying satellite images and predictive modeling, the research identifies a decline in agricultural land and water bodies, along with increasing high-temperature zones through LST. The findings highlight the need for effective policies to support sustainable agriculture and mitigate climate-related risks to food production^40^. The Sundarbans mangrove across India and Bangladesh faced deforestation from 1973 to 2023, but it shows evidence of improving vegetation health, implying its adaptability. The scientific analysis through Landsat and MODIS data specifies a small negative link between vegetation density and surface temperature, indicating that the added elements like water bodies affect temperature, highlighting the importance of strong conservation measures to protect this essential ecosystem^41^. According to Landsat data, the LST and its biophysical parameters, like vegetation, water, and built-up areas, form the Aligarh city’s microclimate. Water bodies and dense vegetation correlate with lower LST, and the urban and bare lands show higher LST. The LU/LC changes match the LST and spectral index trends, validated by Modern-Era Retrospective Analysis for Research and Applications (MERRA) reanalysis data for accurate thermal patterns^42^. The satellite-derived LST with in situ air and surface temperature measurements were compared on the southeastern slope of Lake Baikal. The study found significant differences between the satellite-derived LST and ground-based measurements, enhancing the accuracy of LST estimation through neural networks. The results highlighted the importance of refining satellite LST retrieval methods for better accuracy in the mountainous regions^43^. The GIS-based spatial analyses, statistical methods, and Local Climate Zone (LCZ) classification using Sentinel-2 satellite data examine the thermal environment near Sapanca Lake. Linear regression analyzed the impact of NDVI (Normalized Difference Vegetation Index), MNDWI (Modified Normalized Difference Water Index), and NDBI (Normalized Difference Built-up Index) on LST, revealing NDBI-driven warming across all LCZs, and NDVI and MNDWI have the cooling effects^44^. The impact of several driving factors on LST during the day and night in urban functional blocks (UFBs) in Xi’an, China, was observed. The study analyzed the relationship between LST and factors such as population density, VI, and BUI across different UFBs. The results displayed that the industrial blocks contributed more to daytime LST, and the residential areas had a greater impact at night. Population density and distance from the specific places were the key factors influencing changes in LST^45^. By analyzing the time series Landsat images, the study found a significant increase in the LST, mainly in the areas experiencing rapid development and forest loss in the region of southwestern Ethiopia^46^. The relationship between the LU/LC changes and LST in Amman and Zarqa, Jordan, was investigated effectively. The study identified population density, built-up areas, and vegetation cover as the key factors affecting the LST. The findings highlighted the importance of sustainable planning in reducing the LST and improving urban climate resilience^47^.

The influence of global LST changes on gross primary production (GPP) between 2001 and 2020 was analyzed using various modeling techniques. The findings highlight that machine learning models effectively capture non-linear relationships between LST and GPP with strong statistical significance, where the higher summer temperatures reduce the GPP, and rising winter temperatures promote growth^48^. The Weather Research and Forecasting (WRF) model was integrated with MODIS (Moderate Resolution Imaging Spectroradiometer) satellite data to produce a high-resolution LST map. Tested across eight counties in Iowa, the proposed framework effectively captured the temperature variations under different conditions, significantly reducing bias in WRF simulations and lowering RMSE (Root Mean Squared Error) values^49^. Satellite sensor technology was used to assess deserted wetlands in Pakistan, using Sentinel-2 data (2016–2019) with supervised classification, Tasseled Cap Wetness (TCW), Tasseled Cap Greenness (TCG), and the Normalized Difference Turbidity Index (NDTI). QuickBird images contributed to the Change Detection Index (CDI), while MODIS data provided LST, and an ASTER Digital Elevation Model (DEM) supported the watershed analysis. The results show an increase in water extent in Borith, Phander, Upper Kachura, Satpara, and Rama Lakes, highlighting the necessity for conservation to strengthen ecosystem resilience^50^. The study of Aurangabad city using remote sensing technology shows that the development replaces natural landscapes with impervious surfaces, elevating the LST and affecting thermal comfort. Using ArcGIS software and indices such as IBI (Index-based Built-up Index) and SAVI (Soil-Adjusted Vegetation Index), the research reveals the correlations with the LST indices to guide thermal improvements in semi-arid areas^51^. An analysis of 116 major solar farms worldwide, using MODIS satellite data, shows a decrease in vegetation (measured by Enhanced Vegetation Index, EVI) along with reduced daytime and nighttime LSTs. These effects vary across LU/LC types, seasons, and latitudes in barren areas. The factors such as farm size, type (photovoltaic or concentrating solar power), and local climate influence these outcomes. This information is crucial for sustainable solar energy planning^52^. A worldwide analysis from 2000 to 2024, influencing the MODIS data, examined NDVI-LST relationships across 38,281,647 pixels (20% of Earth), revealing that 80.4% showed strong negative correlations, especially in areas like the western U.S., Brazil, southern Africa, and northern Australia, where higher temperatures reduce vegetation energy. The results demonstrate the vegetation’s role in cooling and its significance for ecosystem flexibility and climate response planning^53^. Integrating the LU/LC spectral indices like NDWI and LST with environmental variables significantly improves the SDM (species distribution models) accuracy using MaxEnt. The results highlight the value of NDWI and multi-source data integration for robust biodiversity modeling and effective conservation planning^54^.

The above studies on LST demonstrate the role of satellite data in assessing development and LU/LC change effects on the environment, mainly in water bodies and vegetation. After careful analysis, we found that the urban heat and dense vegetation misrepresent the LST readings by complicating the water body temperature patterns and lowering monitoring accuracy. The LST struggles to isolate the water’s cooling effect in mixed landscapes by reducing the precision of water temperature tracking. These findings highlight the importance of satellite-based analysis for improving ecological planning. We also observed that environmental changes significantly impact the Earth and its resources. Researchers have observed the increase in the human population and harmful activities that cause the destruction of nature, particularly vegetation and water bodies. The continuous loss has been a concern for society. A challenge in this field involves the calculation of the LST index to perform time-series analyses for environmental change monitoring. The need for highly accurate temporal data to capture the dynamic LU/LC-based LST variations increases the complexity. Furthermore, integrating the LST data with water body identification presents additional challenges, specifically in regions experiencing rapid environmental transformations. To develop early alarm and prevention strategies, technical researchers have emerged to analyze the information about land cover from the past, present, and future using innovative techniques and models. This research addresses these challenges through an efficient ALST-W index, which provides a novel approach to producing time-series maps and enables thorough analyses of LU/LC-based LST changes, contributing to better environmental management strategies.

Inspired by widespread research on the LST, we take on an in-depth investigation in Javadi Hills, India, to evaluate the surface temperature of water bodies and vegetation using LST for different periods. Focusing on the post-COVID period (March 2022 to March 2024), our research highlights the importance of water and vegetation in maintaining ecosystem health and improving overall quality of life. The distinguishing landscape and natural diversity of Javadi Hills provide a unique scenery for this analysis. The authors aim to inform a ground-breaking approach to sustainable environmental management and urban heat reduction in the non-forest-covered region of Javadi Hills. The non-forested areas of the Javadi Hills are selected for ALST-W mapping because their scarce vegetation exposes the direct effect of sunlight on surface temperatures by highlighting the water-cooling effect. Unlike forest-covered areas with dense vegetation that affect thermal readings, these open regions clearly represent water bodies’ heat management. This precision supports satellite data accuracy and strengthens the robust modeling of water body temperature in this naturally vital region. The contributions of this research include:

i. Conducted the ALST-W analysis using the multispectral Landsat 9/8 bands from the non-forest region of Javadi Hills in the years 2020, 2022, and 2024 to study the impact of water bodies on LST.
ii. This research offers useful insights for land managers and forest departments, helping them protect important areas like water bodies and vegetation by clearly analyzing LST changes.

This research work was structured as follows: “Data and methods” outlines the proposed framework of the ALST-W model and details the materials and methodology employed in this research; “Results and observations” presents the experimental outcomes; “Discussion” delivers an in-depth discussion of the findings; and finally, the conclusions are presented in “Conclusion”.

## Data and methods

This section outlines the proposed framework of the ALST-W model and systematically explains the computation of VI, WI, LST, and the ALST-W, providing comprehensive details.

### Proposed work on LU/LC index for analyzing the LST variations on water bodies

**Fig. 1:**
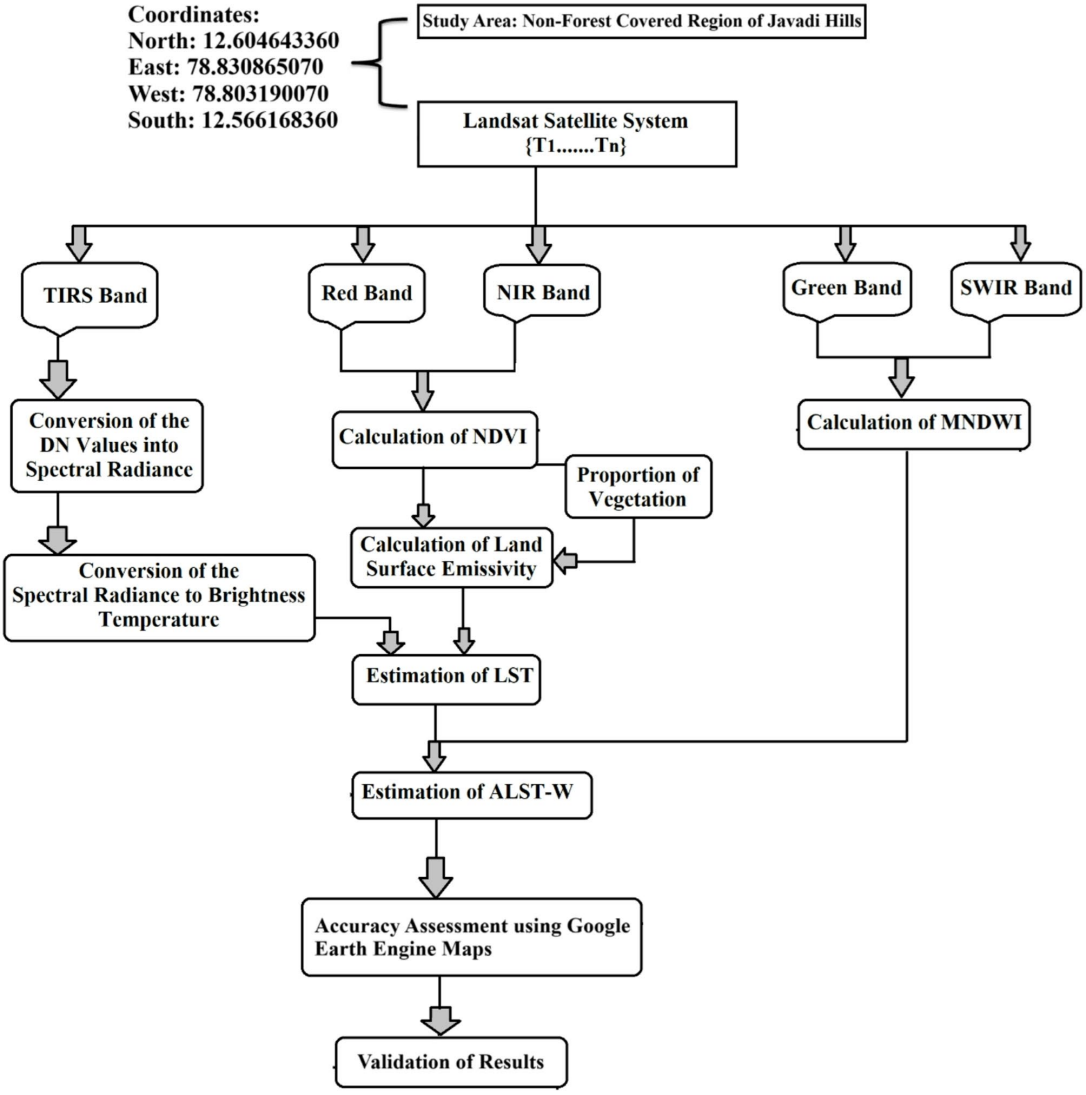
Overview of the ALST-W model framework and workflow.

This research focuses on examining the impact of water bodies on LST in the research region (Non-forest-covered region of Javadi Hills, India) through the ALST-W index using Landsat 8/9 satellite data. The process has been outlined below and shown in Fig. 1. Algorithm 1 provides the complete procedures of our proposed ALST-W index.

i. Landsat satellite data was collected from the non-forest-covered region of the Javadi Hills in India for the years 2020, 2022, and 2024, using various spectral bands.
ii. The Landsat bands like RED, GREEN, NIR (Near Infrared), SWIR (Short-Wave Infrared), and TIRS were pre-processed through radiometric, geometric, and atmospheric corrections to improve data quality.
iii. The ALST-W index was applied to estimate and analyze each year’s data using VI, WI, and LST maps.
iv. Accuracy was measured using the reference GEE map to validate the results and ensure reliable outcomes.
v. The results aim to help land resource planners take necessary actions to prevent environmental degradation and loss.

**Algorithm 1:**
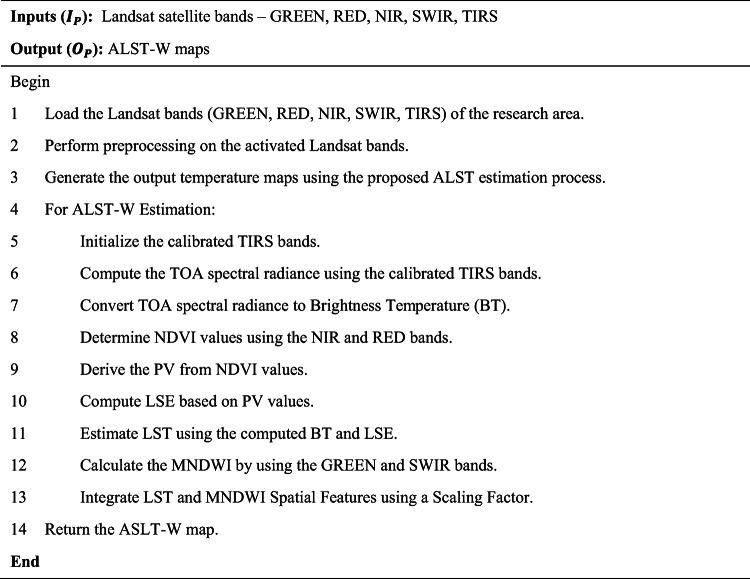
Estimation procedure for ALST-W Index.

### Study area and Landsat data processing

This research calculates the ALST-W for the non-vegetated zones within the Javadi Hills region of Tamil Nadu, India. As part of the Eastern Ghats, the research region spans the forested and non-vegetated territories in the Vellore and Tiruvannamalai districts. The Landsat satellite images of the area are situated between latitudes 12.5 °N to and 12.75 °N and longitudes 78.75 °E and 79.0 °E were acquired for analysis. Table 1 compares the data from Landsat 8 on March 23, 2020, Landsat 8 on March 30, 2022, and Landsat 9 on March 27, 2024, for our research region detected by both satellites. The satellites are equipped with the Operational Land Imager (OLI) and TIRS to measure 11 spectral bands, ranging from Band (B) 1: Coastal/Aerosol to B11: TIRS-2.

**Table 1:** Comparison of Landsat 8 (2020), Landsat 8 (2022), and Landsat 9 (2024) satellite data parameters, spectral bands, and temperature constants.

Parameter	2020 (Landsat 8)	2022 (Landsat 8)	2024 (Landsat 9)
Temporal data	2020-03-24	2022-03-30	2024-03-27
Satellite sensor information	Landsat 8 - OLI and TIRS	Landsat 8 - OLI and TIRS	Landsat 9 - OLI and TIRS
Cloud cover	0.30%	1.51%	1.24%
Spectral bands			
B1	Coastal/aerosol		
B2	Blue		
B3	Green		
B4	Red		
B5	NIR		
B6	SWIR-1		
B7	SWIR-2		
B8	Panchromatic		
B9	Cirrus		
B10	TIRS-1		
B11	TIRS 2		
Temperature			
RADIANCE_MULT_BAND_10	3.3420E-04	3.3420E-04	3.8000E-04
RADIANCE_ADD_BAND_10	0.10000	0.10000	0.10000
K1_CONSTANT_BAND_10	774.8853	774.8853	799.0284
K2_CONSTANT_BAND_10	1321.0789	1321.0789	1329.2405
RADIANCE_MULT_BAND_11	3.3420E-04	3.3420E-04	3.4900E-04
RADIANCE_ADD_BAND_11	0.10000	0.10000	0.10000
K1_CONSTANT_BAND_11	480.8883	480.8883	475.6581
K2_CONSTANT_BAND_11	1201.1442	1201.1442	1198.3494
Weather			
SUN_ELEVATION	61.28314085	62.50589922	61.99915294
SUN_AZIMUTH	111.23799360	107.36488645	109.16898399
Source	USGS, United States of America (https://earthexplorer.usgs.gov)		

**Fig. 2:**
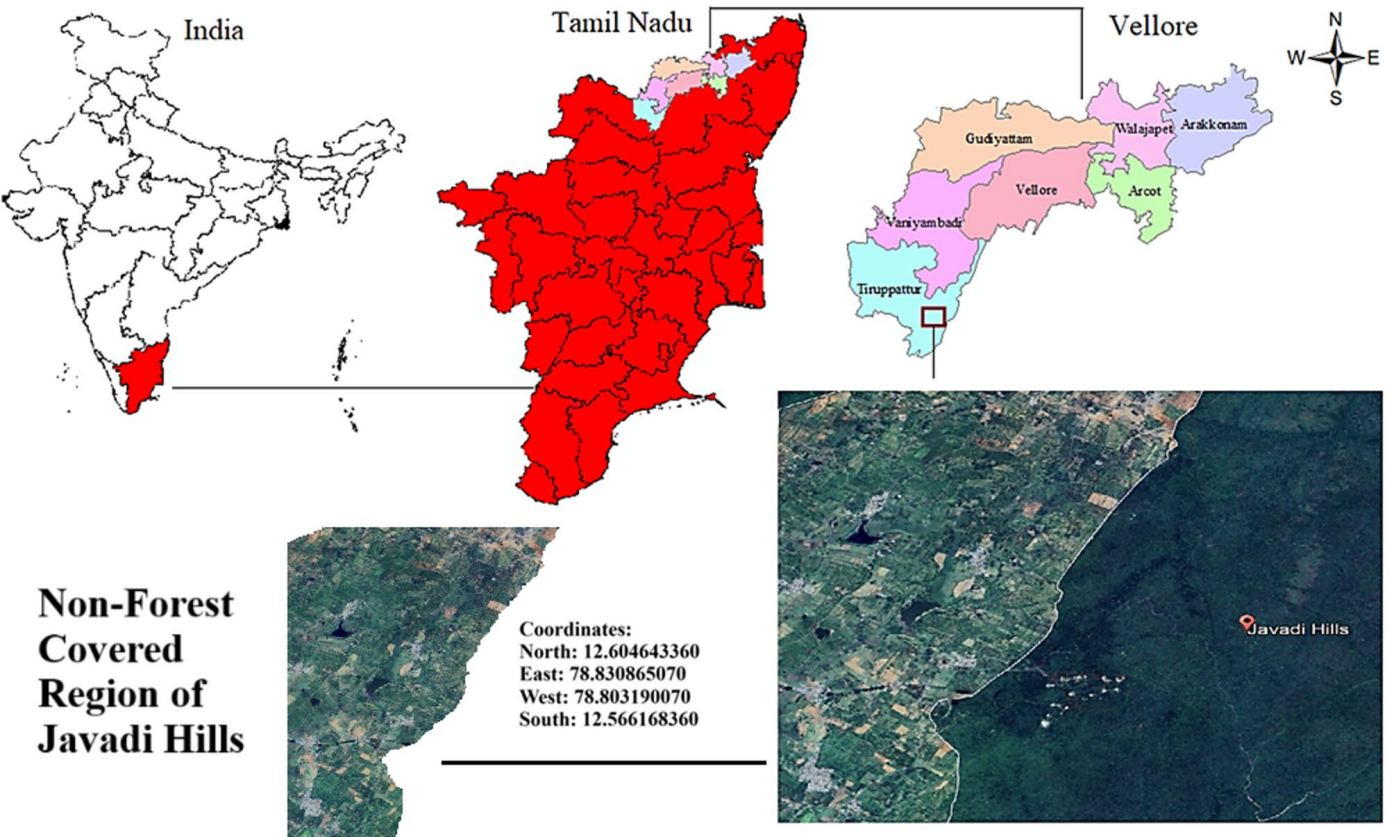
Thematic location map – research region (non-forest covered region of Javadi Hills). Maps were generated using QGIS version 3.40.0 (https://www.qgis.org), Google Earth Engine (https://www.google.com/earth/), and shapefiles sourced from DIVA GIS (https://diva-gis.org/). The maps were finalized using image editing software (Microsoft Paint for Windows).

**Fig. 3:**
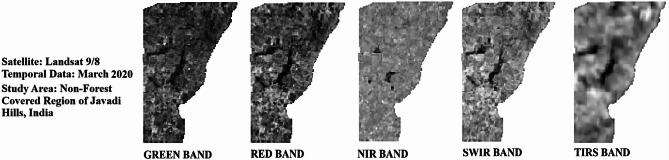
Landsat bands for non-forest covered region of Javadi Hills, 2020: pre-processed data.

**Fig. 4:**
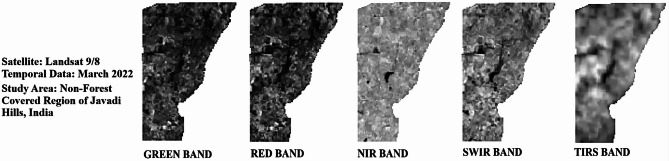
Landsat bands for non-forest covered region of Javadi Hills, 2022: pre-processed data.

**Fig. 5:**
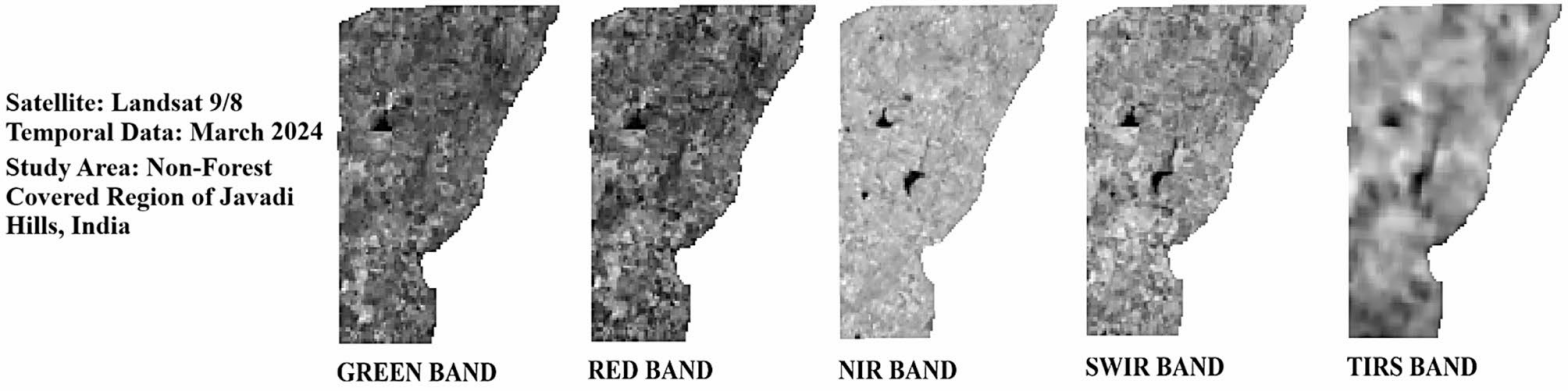
Landsat bands for non-forest covered region of Javadi Hills, 2024: pre-processed data.

Cloud cover of the Landsat bands was at 0.30% in 2020, 1.51% in 2022, and 1.24% in 2024, showing slightly clearer skies with good visibility for satellite bands without clouds. Temperature-related values for TIRS bands (B10 and B11) indicate the differences in radiance multipliers, such as 3.3420E-04 for the years 2020 and 2022 and 3.8000E-04 for 2024 in Band 10, and thermal constants, like K1 for Band 10 at 774.8853 in 2020 and 2022 and 799.0284 in 2024. The additive factors stayed at 0.10000 for all the years. Weather data shows a sun elevation of 61.28° in 2020, 62.51° in 2022, and 61.99° in 2024, with sun azimuth shifting from 111.27° in 2020 to 107.36° in 2022 to 109.17° in 2024 by reflecting the small changes in solar position. All information was obtained from the USGS (United States Geological Survey), available at https://earthexplorer.usgs.gov, ensuring a reliable source for these measurements. A thematic spatial map of the research region was extracted using advanced geospatial software, QGIS version 3.40.0 (https://www.qgis.org), and GEE (https://www.google.com/earth/), with shapefiles sourced from DIVA-GIS (https://diva-gis.org/), which provides free spatial data. Figure 2 illustrates the spatial layout for the non-vegetated sectors of the research region. The atmospheric, radiometric, and geometric corrections were employed to improve the clarity of the raw satellite image. Figure 3 (March 2020), Fig. 4 (March 2022), and Fig. 5 (March 2024) provide the pre-processed image across multiple spectral bands like Green, Red, NIR, SWIR, and TIRS to assess the variations in LU/LC and thermal properties over the three years. The key difference between the three figures was the temporal gap, with Fig. 3 providing the baseline conditions of 2020 and Figs. 4 and 5 showing the changes by 2022 and 2024, enabling a comparative analysis of environmental changes. These images collectively deliver comprehensive information for assessing the changing aspects of the non-forest region in the Javadi Hills.

### Estimation of the proposed ALST-W index

The Land Surface Temperature (LST) index locates regions with elevated and reduced thermal variations on the Earth’s surface within a defined geographic area. The surface thermal outline quantifies the reflected radiative energy, enabling precise LST computation. The TIRS band captures a composite of high and low thermal signatures^52,55,56^. TIRS spectral data from Landsat 9/8 platforms were employed to calculate LST in our analysis. Equation (1) to (7) demonstrate the computations for the TIRS bands of the Landsat 9/8 systems.

1 $$\:{TL}_{{\uplambda\:}}=ML*\:QCAL+AL-\:{O}_{i}$$

Equation (1) illustrates the process for converting the Top of Atmosphere (TOA) spectral radiance ($\:{TL}_{{\uplambda\:}})$ for the TIRS bands, using the radiance rescaling coefficient ($\:ML$) in units of $\:(Watts/{(m}^{2}*{sr}^{2}*\:\mu\:m))$. The quantized calibrated pixel was denoted by $\:QCAL$, with $\:AL$ representing the radiance offset rescaling factor. Furthermore, the correction term $\:{O}_{i}$ for the Landsat 8/9 TIRS band has been incorporated into the calculation.

2 $$\:{BT}_{P}=\:\frac{K2}{\text{ln}\left(\frac{K1}{{TL}_{{\uplambda\:}}}+1\right)}-273.15$$

Equation (2) details the method for transforming TOA brightness temperature ($\:{BT}_{P}$) from spectral radiance data into Celsius ($\:C$). This conversion process employs calibration parameters $\:K1\:and\:K2$, which are measured in$\:\:(Watts/{(m}^{2}*{sr}^{2}*\:\mu\:m))$. The K1 and K2 values for the Landsat 8 TIRS band are 774.8853 and 1321.0789, respectively, whereas for the Landsat 9 TIRS band, they are 799.0284 and 1329.2405.

3 $$\:NDVI=\frac{\left(NIR-RED\right)}{(NIR+RED)}$$

4 $$\:PV={\left(\right(NDVI-\:{NDVI}_{min})/\:({NDVI}_{max}\:-\:{NDVI}_{min}\:\left)\right)}^{2}$$

5 $$\:E=0.004*PV+0.986$$

The Land Surface Emissivity ($\:E$) was determined using $\:NDVI\:\left(Normalized\:Difference\:Vegetation\:Index\right)$ values from Eq. (3), where $\:PV$ stands for vegetation fraction (Proportion of Vegetation), and $\:{NDVI}_{max}$ and $\:{NDVI}_{min}$ refer to the maximum and minimum reflectance values from the Landsat 8/9 $\:NDVI$ image. In Eq. (3), $\:NIR,$ and RED correspond to the reflectance measurements from the RED and NIR spectral bands of the Landsat satellite system. The calculations for $\:PV$ and $\:E$ are outlined in Eqs. (4) and (5). The LST computation of Landsat 8/9 follows the results from Eqs. (1) to (5), using the expression outlined in Eq. (6).

6 $$\:LST=\:\frac{{BT}_{P}}{\left(1+\:\left(\raisebox{1ex}{$\lambda\:*{BT}_{P}$}\!\left/\:\!\raisebox{-1ex}{$c2$}\right.\:\right)*\:{ln}\left(E\right)\right)}$$

7 $$\:c2=\raisebox{1ex}{$pk*vl$}\!\left/\:\!\raisebox{-1ex}{$bc$}\right.$$

where $\:{\uplambda\:}$ denotes the wavelength of the emitted radiation, Planck’s constant ($\:pk)$ has a value of $\:6.626\text{*}{10}^{-34}\:\text{J}\:\text{s}$, the velocity of light ($\:vl$) is $\:2.998\:\text{*}\:108\:\text{m}/\text{s}$, and the Boltzmann constant ($\:bc$) is $\:1.38\:\text{*}\:{10}^{-34}\:\text{J}\text{K}$. The importance of evaluating LU/LC metrics lies in revealing the unique value ranges associated with each LU/LC category. Indicators like VI and WI are employed to identify features such as high and low vegetation and water bodies. Each spectral band from the satellite data determines its specific LU/LC characteristics^57–59^. In our work, MNDWI was applied to the non-forest areas of our research area because it includes built-up regions. We chose MNDWI as it improves water body detection in these areas, where distinguishing water from buildings or vegetation is more difficult. The NDWI (Normalized Difference Water Body Index) is better suited for detecting water in natural, vegetated environments. Equation (8) determines these MNDWI metrics.

8 $$\:MNDWI=\frac{\left(GREEN-SWIR\right)}{(GREEN+SWIR)}$$

In Eq. (8), $\:SWIR,$ and $\:GREEN$ corresponds to the reflectance measurements from the SWIR, and GREEN spectral bands of the Landsat satellite system. We have proposed a new estimation of the ALST-W index using the results of LST, and MNDWI. The proposed index was applied to analyze water bodies, vegetation, and barren land temperature values effectively. Equation (9) proposes the impact of water bodies on the LST

9 $$\:ALS{T}_{W}=LST-\left(scaling\:factor*\:MNDWI\right)$$

**Fig. 6:**
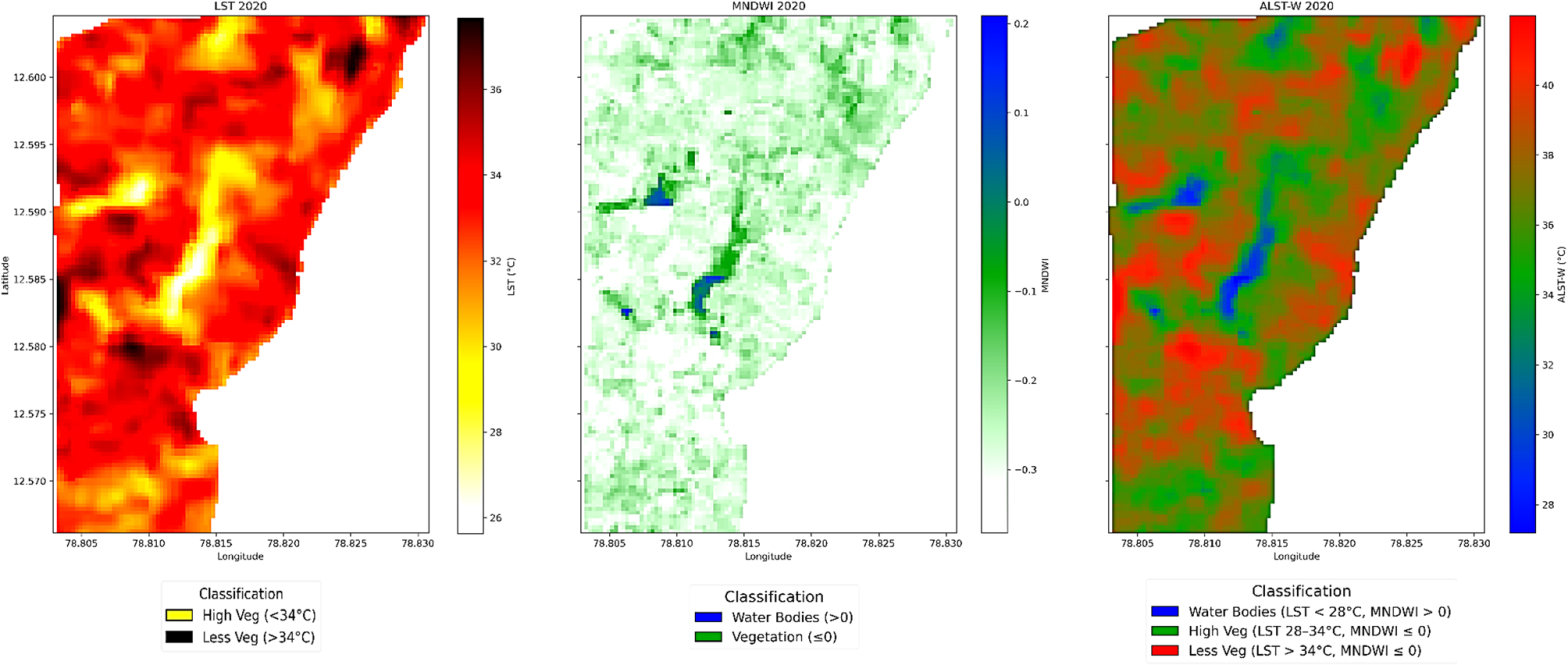
LST, MNDWI, and ALST-W maps, 2020 (non-forest covered region of Javadi Hills, India). Results were generated using JupyterLab version 4.2.5 (https://jupyter.org/) with Python version 3.12.3 (https://www.python.org/downloads/release/python-3123/), accessed through Anaconda Navigator version 2.6.5 (https://www.anaconda.com/products/navigator).

**Fig. 7:**
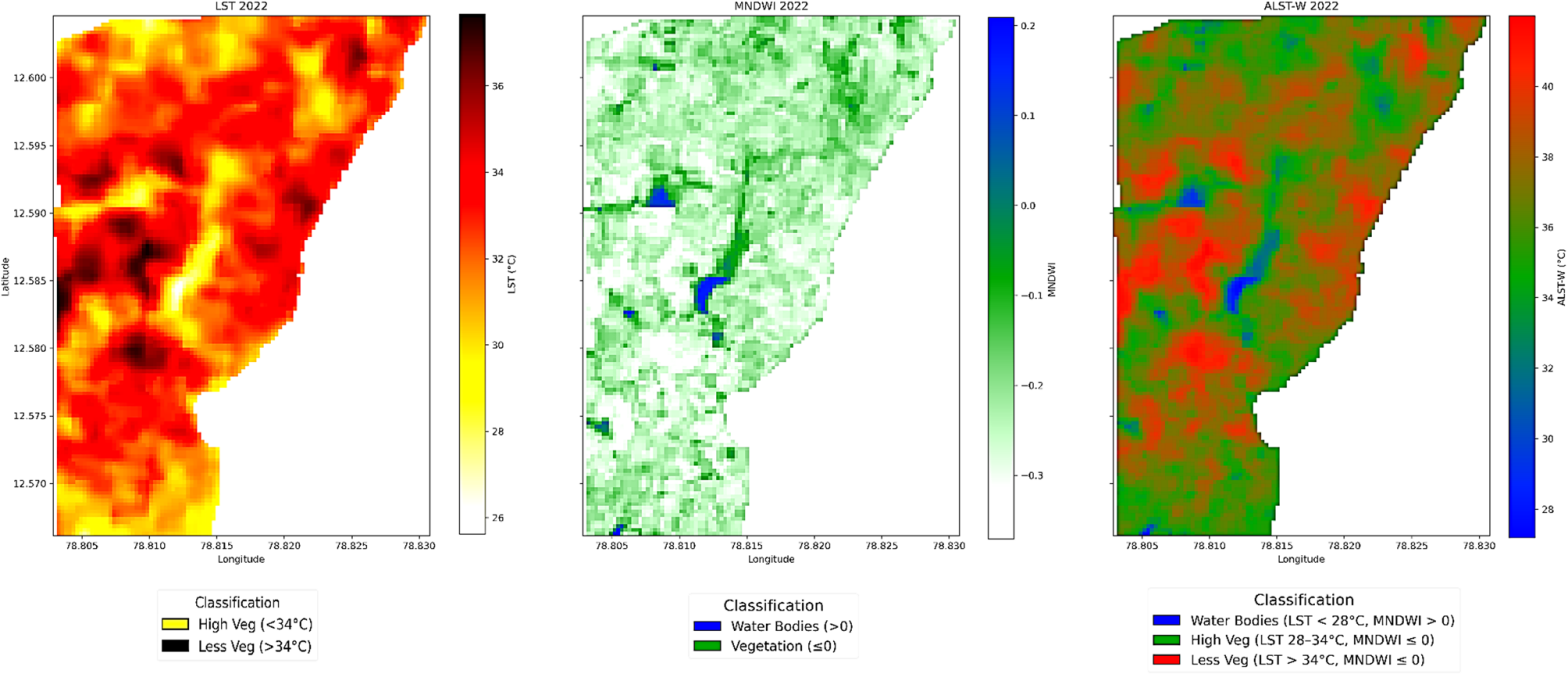
LST, MNDWI, and ALST-W maps, 2022 (non-forest covered region of Javadi Hills, India). Results were generated using JupyterLab version 4.2.5 (https://jupyter.org/) with Python version 3.12.3 (https://www.python.org/downloads/release/python-3123/), accessed through Anaconda Navigator version 2.6.5 (https://www.anaconda.com/products/navigator).

**Fig. 8:**
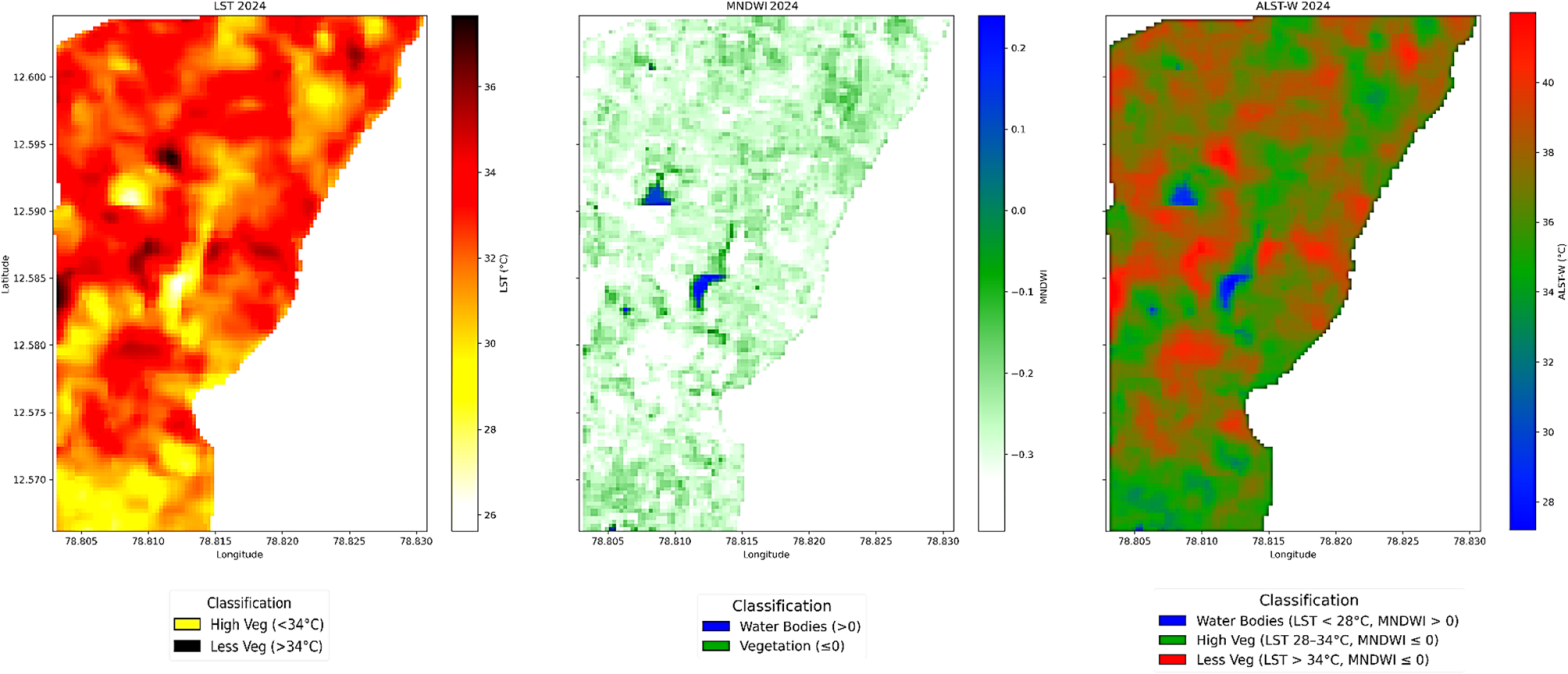
LST, MNDWI, and ALST-W maps, 2024 (non-forest covered region of Javadi Hills, India). Results were generated using JupyterLab version 4.2.5 (https://jupyter.org/) with Python version 3.12.3 (https://www.python.org/downloads/release/python-3123/), accessed through Anaconda Navigator version 2.6.5 (https://www.anaconda.com/products/navigator).

The relevance of our research is to analyse water body effects on the Earth’s surface interactions, and thermal dynamics. We achieved the results through MNDWI (Eq. 8) and LST (Eq. 6) estimation. Equation (9) delivers the proposed equation for finding the ALST-W index. Table 2 outlines the influence of the available VI, WI, and LST indices, with the proposed ALST-W index, on evaluating the environmental conditions. The output maps are shown in Figs. 6 and 7, and 8.

**Table 2:** Contribution of existing and proposed indices to environmental assessment of the research region.

Spatial Maps	Uses
NDVI^44^	Measure the vegetation health and density in urban and non-urban locations, although its effectiveness may differ based on the specific landscape
NDWI^54^	Identifies the water bodies in natural and vegetated regions
MNDWI^44^	Differentiates the Water Bodies from high and low Vegetation, mainly in urban areas
LST^55^	Display the mixture of high and low vegetation soil temperatures
Proposed ALST-W [Ours]	Display the mixture of water bodies and high and low vegetation soil temperatures

### LST classification and threshold determination

In this research, we performed the LST classification and threshold determination to accurately separate LU/LC classes associated with the temperature values by supporting precise environmental assessment and planning. The proposed ALST-W index enhances the temperature-based analysis. Three LST-based LU/LC classes (water bodies, low vegetation, and high vegetation) were classified using the proposed ALST-W index map. The scaling factor was applied to normalize the temperature values for accurate extraction. The ALST-W index fine-tunes the raw LST by subtracting a scaled MNDWI factor, efficiently lowering the temperature estimation in areas with higher water content (positive MNDWI) and raising it in low vegetated areas (negative MNDWI). The scaling factor in this proposed index adjusts the impact of MNDWI on LST to generate ALST-W, mainly to justify their distinct units and ranges. The LST was measured in degrees Celsius (°C), whereas MNDWI is a dimensionless value between − 1 and + 1. The scaling factor bridges this gap by strengthening the MNDWI’s effect into a temperature-compatible scale. In this research work, we set the scaling factor to 10. For this research, we executed a scaling factor of 10 to adjust MNDWI’s range (− 1 to + 1) into a ± 10 °C temperature modification for ALST-W by effectively connecting the moisture levels to LST variations. In 2020, LST varied between 28.08 °C and 38.66 °C (with a mean of 34.47 °C) and MNDWI between − 0.37 and 0.20 (with a mean of – 0.26), providing an ALST-W range of 27.18–41.99 °C (with a mean of 37.11 °C). In 2022, LST covered 27.97 °C to 34.30 °C (with a mean of 31.60 °C), and MNDWI spanned − 0.36 to 0.18 (with a mean of − 0.23), resulting in an ALST-W range of 26.47 °C to 37.79 °C (with a mean of 33.96 °C). In 2024, LST ranged from 25.62 to 37.66 °C (with a mean of 32.18 °C) and MNDWI from − 0.39 to 0.23 (with a mean of − 0.27), producing an ALST-W range of 23.51–40.73 °C (with a mean of 34.93 °C). These statistics determine that a factor of 10 generates ALST-W changes that correspond closely to real-world temperature differences driven by the cooler wet zones (MNDWI > 0).

We performed a sensitivity analysis by testing the different scaling values from 5 to 15 to measure their effect on the configuration between LST and MNDWI patterns. The reference maps obtained from GEE ensure accuracy and consistency of the proposed ALST-W map. The analysis revealed that a scaling factor of 10 presented the best stability by reducing distortion while sustaining the spatial thermal gradients. This factor consistently achieved the highest visual and statistical arrangement between LST and water body description by supporting its selection for the ALST-W index. This work cross-validated the ALST-W results using high-resolution imagery from GEE to evaluate accuracy by ensuring the consistency of the LU/LC classes, like water bodies, high vegetation, and low vegetation. The GEE served as a reference map to systematically analyze and validate the threshold values by enhancing the robustness of the results. The threshold values were determined by the ranges of ALST-W, with high-resolution images from GEE as a reference. The approach involved aligning the ALST-W classified pixels with visually evaluated reference points in GEE to ensure consistency with the LU/LC classes. The specific thresholds defined are as follows: ALST-W below 28.0 °C for water bodies, ALST-W between 28.0 and 34.0 °C for high vegetation, and ALST-W above 34.0 °C for low vegetation. These parameters resulted in a reliable and effective classification framework. A histogram was also employed to visually represent the ALST-W data distribution by providing a clear and instinctive framework for understanding the results. “Results and observations” provides a detailed view of the results and observations.

### Experimental configuration

The experiments represent the effectiveness of the ALST-W index in analyzing the water body temperature variations for 2020, 2022, and 2024 in the non-forest-covered region of the Javadi Hills, India. The pre-processed satellite images from Landsat 9/8 were analyzed using JupyterLab version 4.2.5 (https://jupyter.org/) with Python version 3.12.3 (https://www.python.org/daownloads/release/python-3123/), accessed through Anaconda Navigator version 2.6.5 (https://www.anaconda.com/products/navigator), to extract and analyze the appropriate geospatial data. The computational setup included an Intel(R) Core (TM) i5-13420 H (2.10 GHz) processor, ensuring efficient and precise data processing. The ALST-W index provides the impact of water bodies on LST. This configuration ensured a methodical approach to data acquisition, processing, and analysis by aiding in consistent results.

## Results and observations

This section outlines our study’s results and observations. We present a concise evaluation of the ALST-W index, including the corresponding LU/LC VI, WI, and LST values extracted from the research region’s time series data. Our research comprehensively analyzed temperature values for water bodies and areas with high and low vegetation. Figures 6 and 7, and 8 display three output maps of the research region for 2020, 2022, and 2024, showing the LST, MNDWI, and the proposed ALST-W with visually classified descriptions. The facts displayed in the figures correspond to the information provided in Tables 3, 4 and 5, which detail the statistical measures of LST, MNDWI, and the proposed ALST-W for 2020, 2022 and 2024. Table 3 provides the information on LST, MNDWI, and ALST-W for the year 2020. The average LST positions represent the typical surface temperature at 34.477089 °C, with a standard deviation of 1.516327, pointing to relatively constant conditions. The MNDWI, ranging from − 0.371 to 0.209, indicates the presence of water bodies, with a mean of -0.264, signifying a most arid region with a standard deviation of 0.055. The proposed ALST-W shows an average of 37.118458 °C and a standard deviation of 1.946654 by reflecting the weighted temperature variances with values ranging from 27.188206 °C to 41.993477 °C.

**Table 3:** Statistical summary of LST, MNDWI, and ALST-W for the year 2020 (Non-Forest covered region of Javadi hills, India).

Statistics	Range		
	LST (°C)	MNDWI (– 1 to + 1)	ALST-W (°C)
Mean	34.477089	– 0.264137	37.118458
Standard deviations	1.516327	0.055326	1.946654
Minimum	28.083399	– 0.371057	27.188206
Median	34.598206	– 0.272028	37.315006
Maximum	38.665051	0.209133	41.993477

**Table 4:** Statistical summary of LST, MNDWI, and ALST-W for the year 2022 (Non-Forest covered region of Javadi hills, India).

Statistics	Range		
	LST (°C)	MNDWI (– 1 to + 1)	ALST-W (°C)
Mean	31.600229	– 0.236148	33.961712
Standard deviations	0.920414	0.062897	1.409935
Minimum	27.977360	– 0.367314	26.477661
Median	31.621685	– 0.243332	34.040524
Maximum	34.301399	0.188916	37.793255

**Table 5:** Statistical summary of LST, MNDWI, and ALST-W for the year 2024 (Non-Forest covered region of Javadi hills, India).

Statistics	Range		
	LST (°C)	MNDWI (– 1 to + 1)	ALST-W (°C)
Mean	32.186798	– 0.274907	34.935871
Standard deviations	1.551967	0.058262	1.958531
Minimum	25.625717	– 0.394414	23.514002
Median	32.324749	– 0.279656	35.124607
Maximum	37.663406	0.239928	40.736485

**Fig. 9:**
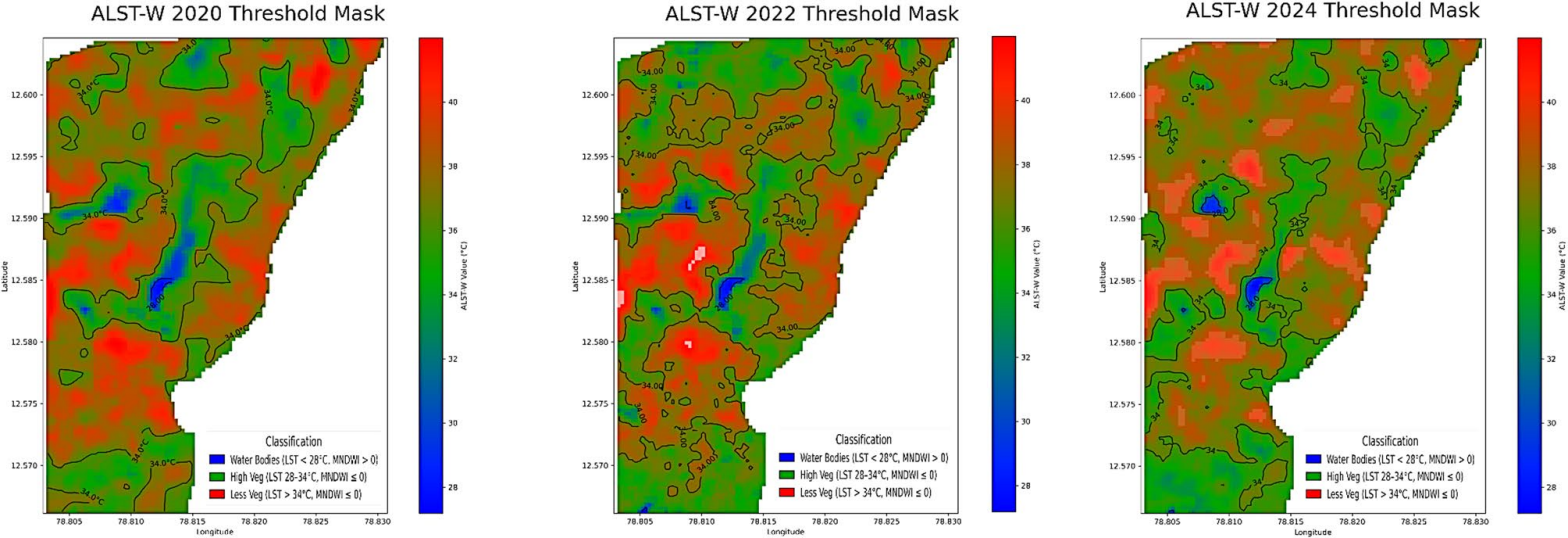
ALST-W threshold masks for 2020, 2022 and 2024. Results were generated using JupyterLab version 4.2.5 (https://jupyter.org/) with Python version 3.12.3 (https://www.python.org/downloads/release/python-3123/), accessed through Anaconda Navigator version 2.6.5 (https://www.anaconda.com/products/navigator).

**Fig. 10:**
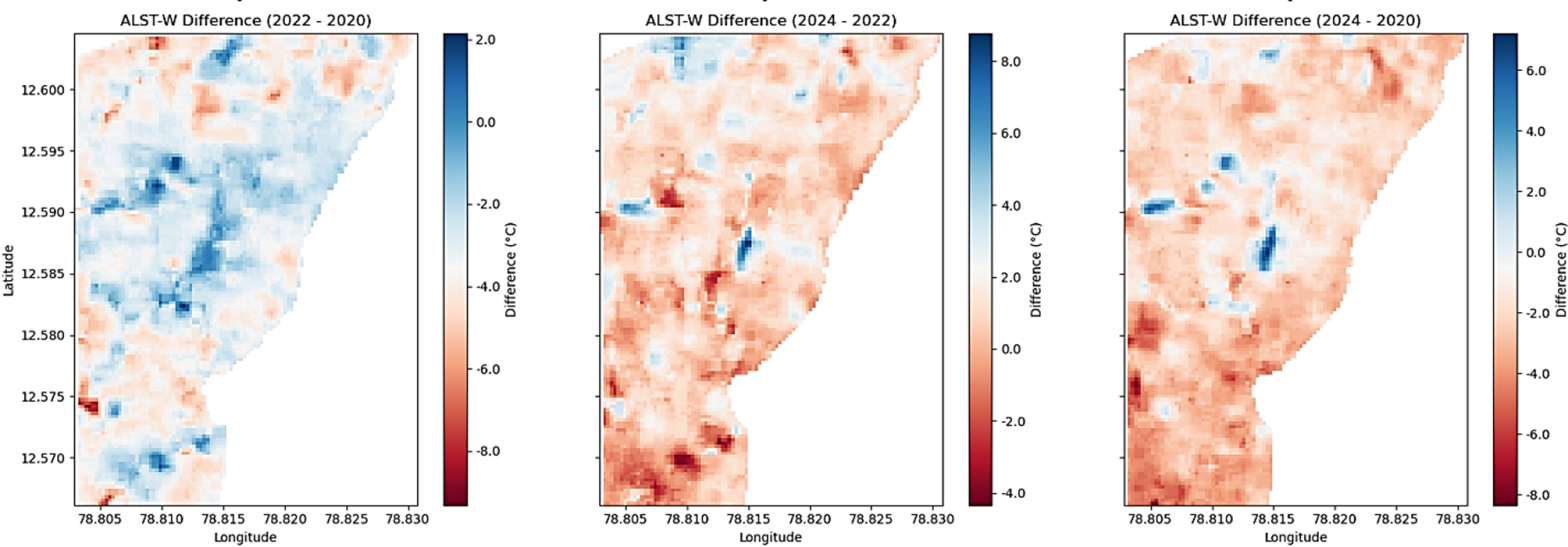
ALST-W difference maps from 2020 to 2024. Results were generated using JupyterLab version 4.2.5 (https://jupyter.org/) with Python version 3.12.3 (https://www.python.org/downloads/release/python-3123/), accessed through Anaconda Navigator version 2.6.5 (https://www.anaconda.com/products/navigator).

Table 4 provides the information on LST, MNDWI, and ALST-W for 2022. The average LST positions represent the typical surface temperature at 31.600229 °C, displaying a minor decrease from 2020, with a standard deviation of 0.920414, pointing to relatively constant conditions. The MNDWI, ranging from − 0.367314 to 0.188916, indicates the presence of water bodies, with a mean of – 0.236148, signifying a most arid region with a standard deviation of 0.062897. The proposed ALST-W shows an average of 33.961712 °C and a standard deviation of 1.409935 reflecting the weighted temperature variances with values ranging from 26.477661 to 37.793255 °C. Table 5 provides the information on LST, MNDWI, and ALST-W for 2024. The data provides an average LST of 32.186798 °C, displaying a minor increase from 2022 and a higher standard deviation of 1.551967, demonstrating the greater variations in temperature. The MNDWI range extends from − 0.394414 to 0.239928, with a mean of – 0.274907, implying a drier pattern compared to 2022, and a standard deviation of 0.058262 specifies the stable moisture conditions. The proposed ALST-W records a mean of 34.935871 °C, higher than in the year 2022, with a standard deviation of 1.958531 and a range between 23.514002 and 40.736485 °C, reflecting the broader temperature swings. ALST-W index of 2020, 2022, and 2024 revealed variations in surface temperatures and moisture levels. Mean LST and ALST-W values specified a temperature decline from 2020 to 2022, followed by a minor rise in 2024. MNDWI consistently showed arid conditions, with 2024 marking the driest year. Increasing standard deviations for LST and ALST-W from 2022 to 2024 suggest growing temperature variability, while stable MNDWI standard deviations indicate consistent moisture levels despite the drier trend observed in 2024. Table 6 summarizes the statistical analysis of LST, MNDWI, and ALST-W metrics for the non-forest-covered region of Javadi Hills from 2020 to 2024,including the interpretations based on class-specific thresholds. The impact of human activity and climatic variations was a cause of change in the ALST-W variations of Javadi Hills. Minimal ALST-W values correspond to water bodies. Moderate-level values correspond to vegetated zones, and higher values correspond to low vegetation. A comparison was conducted with the GEE reference image to validate the results. The clear overview of the environmental conditions in the non-forest-covered region of Javadi Hills was reflected, presented, and compared in this work for three different years: March 2020, March 2022, and March 2024. A comparative assessment of the three distinct years (2020, 2022, and 2024) enabled us to evaluate changes in LU/LC of three different classes (water, high, and low vegetation) through the proposed thermal features. We have examined and demonstrated these findings through the threshold mask in Fig. 9. The areas below 28 °C indicate the water bodies, with reductions indicating possible drying. Temperatures between 28 and 34 °C represent the healthy vegetation, which is useful in crop and forest monitoring. Regions above 34 °C reflect sparse vegetation or degraded land, with increasing signs of rising heat. This information assists the concerned government officials, forest departments, and farmers in managing the resources effectively. Figure 10 presents the difference between the ALST-W maps for 2020, 2022, and 2024 across regional and local levels. Temperature contours outline the zones of uniform heat, with cooler regions (< 28.0 °C) tied to water bodies and dense vegetation (28.0–34.0 °C) and hotter regions (> 34.0 °C) reflecting the low vegetation or urban areas. The difference maps (2022–2020, 2024–2022, 2024–2020) display warming patterns (> 2 °C) in areas with vegetation loss or urban growth and cooling patterns (<-2 °C) where water bodies or greenery have expanded. Contours at ± 2 °C and ± 5 °C mark areas of notable temperature changes, pointing to the key locations for focused actions such as afforestation, irrigation, or conservation to mitigate warming and enhance cooling areas.

**Table 6:** Statistical summary of LST, MNDWI, and ALST-W metrics across years (2020–2024) with class-based threshold interpretations.

Statistic	Year	LST (°C)	MNDWI (– 1 to + 1)	ALST-W (°C)	Explanation
Mean	2020	34.477089	– 0.264137	37.118458	2020 displayed the highest average ALST-W, with most areas exceeding 34.0 °C, representing the dominant low vegetation cover and limited water presence
	2022	31.600229	– 0.236148	33.961712	In 2022, the mean ALST-W dropped near the 34.0 °C threshold, marking a shift toward increased high vegetation coverage and some water body presence
	2024	32.186798	– 0.274907	34.935871	2024 experiences a slight rise above 34.0 °C in mean ALST-W, indicating a return of low vegetation areas and reduced water body extent compared to 2022
Standard deviation	2020	1.516327	0.055326	1.946654	Moderate variability in 2020 reflects a mix of ALST-W zones, though dominated by low vegetation
	2022	0.920414	0.062897	1.409935	Lower variability in 2022 suggests a more uniform distribution of high vegetation and transitional zones
	2024	1.551967	0.058262	1.958531	Increased variability in 2024 implies a more scattered distribution of ALST-W values across all three classes
Minimum	2020	28.083399	– 0.371057	27.188206	Minimum ALST-W in 2020 hardly enters the water body threshold (< 28 °C), suggesting very limited cooler zones
	2022	27.977360	– 0.367314	26.477661	2022 shows a stronger presence of water bodies, with minimum ALST-W well below the 28.0 °C threshold
	2024	25.625717	– 0.394414	23.514002	2024 had the lowest minimum ALST-W, indicating a broader extent or the emergence of cooler water body zones
Median	2020	34.598206	– 0.272028	37.315006	The 2020 median ALST-W value was above 34.0 °C, confirming the dominance of the low vegetation class
	2022	31.621685	– 0.243332	34.040524	The 2022 median ALST-W value aligns closely with the upper high vegetation threshold, showing mixed zones
	2024	32.324749	– 0.279656	35.124607	The 2024 median slightly above 34.0 °C indicates a tilt toward low vegetation, but close to the transitional range
Maximum	2020	38.665051	0.209133	41.993477	Maximum ALST-W in 2020 far exceeds the 34.0 °C threshold by confirming intense low vegetation
	2022	34.301399	0.188916	37.793255	Slightly lower than 2020, where the max ALST-W in 2022 still reflects dominant low vegetation zones, but with reduced intensity
	2024	37.663406	0.239928	40.736485	Max ALST-W in 2024 rose again, suggesting a return of extreme heat in low vegetation zones

**Fig. 11:**
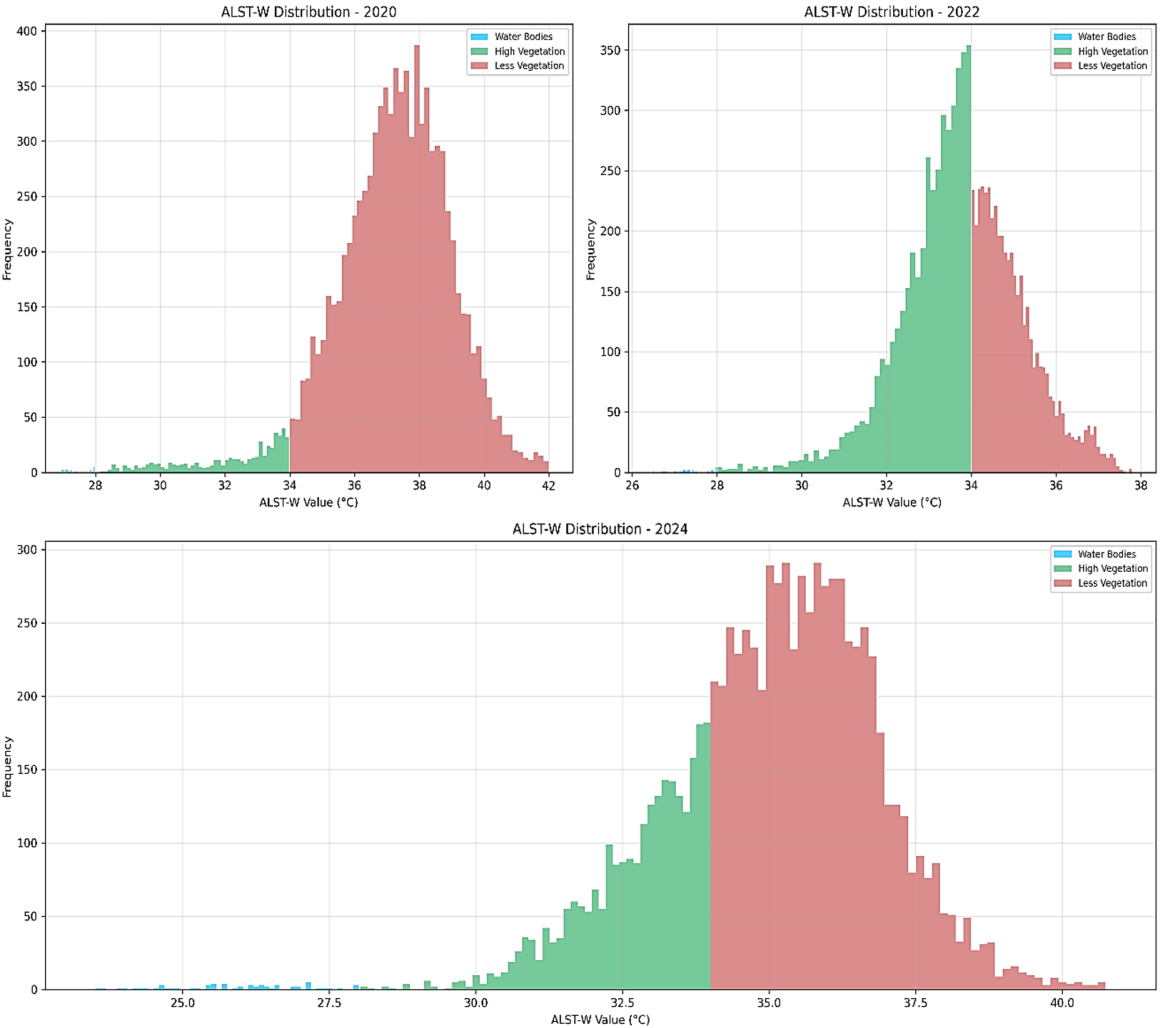
ALST-W distribution comparison for 2020, 2022, and 2024 through Histogram chart. Results were generated using JupyterLab version 4.2.5 (https://jupyter.org/) with Python version 3.12.3 (https://www.python.org/downloads/release/python-3123/), accessed through Anaconda Navigator version 2.6.5 (https://www.anaconda.com/products/navigator).

**Fig. 12:**
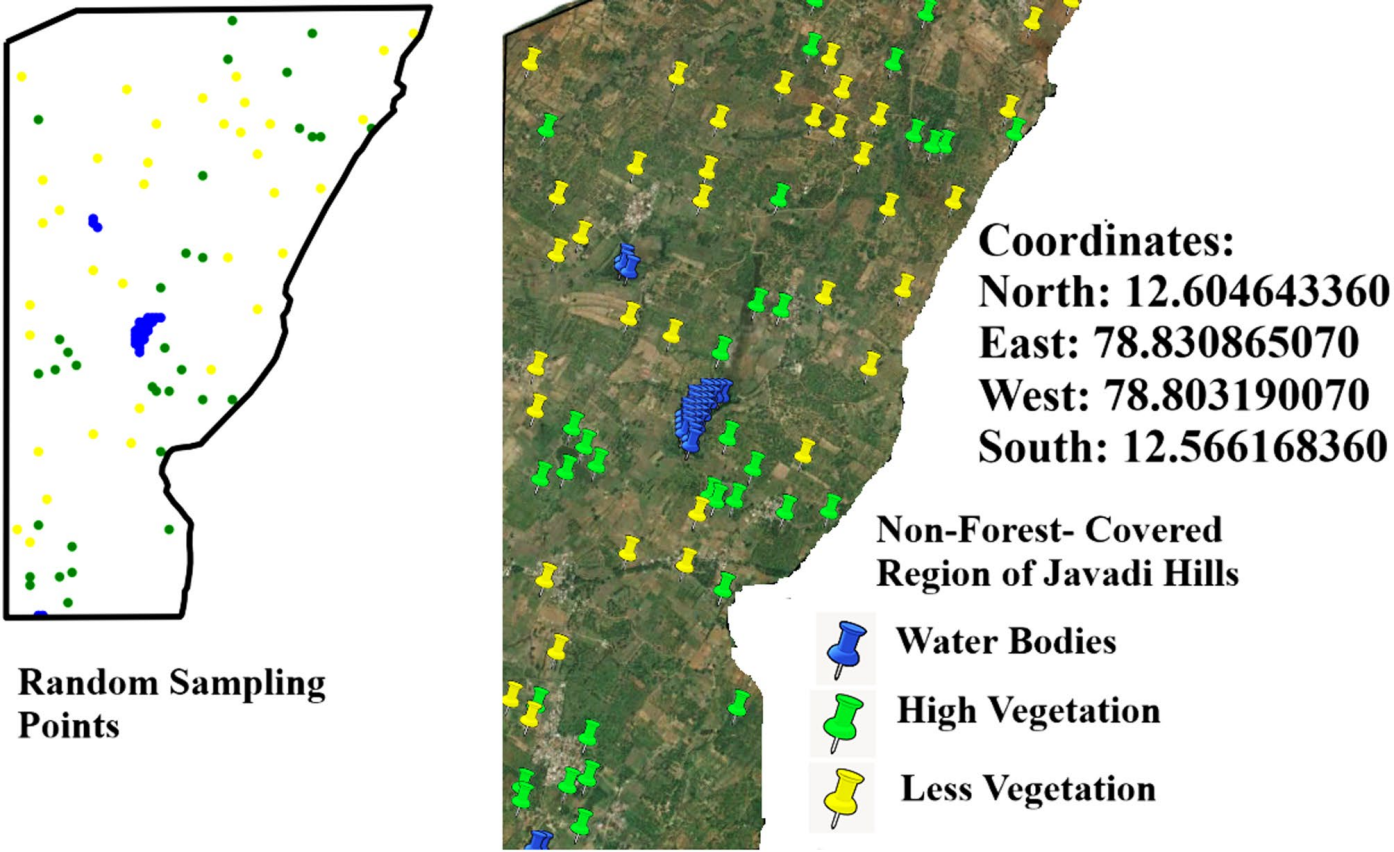
GEE-based reference map showing random LU/LC (Land Use/Land Cover) samples. The samples were collected and generated using Google Earth Engine (https://www.google.com/earth/).

**Fig. 13:**
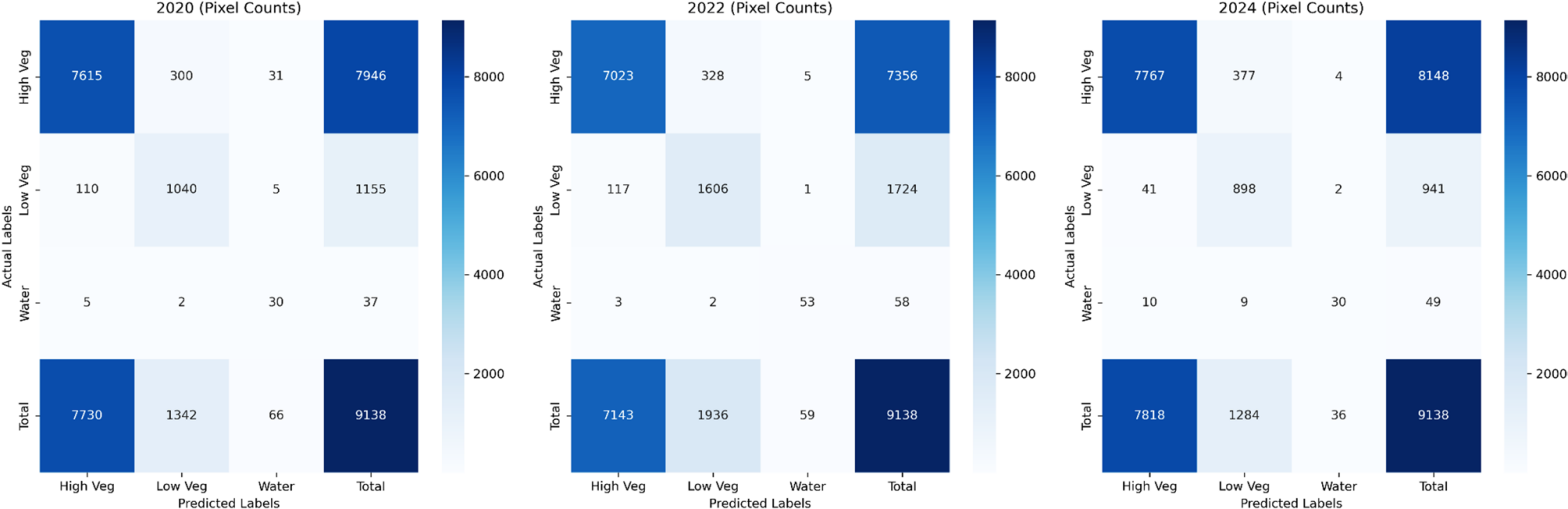
Visualization of confusion matrices based on overall classified pixel count with actual vs. predicted LU/LC classes for 2020, 2022, and 2024.

Figure 11 visualizes the histogram chart of ALST-W values. The histogram evaluates the distribution of ALST-W values (°C) for 2020, 2022, and 2024 across the water bodies, high vegetation, and low vegetation. For the time series data from 2020 to 2024, the low vegetation increases and shifts toward higher temperatures, while high vegetation slightly declines. The water bodies remain consistently limited throughout all years, indicating minimal change in cooler zones. Table 7 presents the spatial distribution of the LU/LC classes derived from ALST-W thermal histogram analysis conducted between 2020 and 2024. This study validated the ALST-W results by cross-referencing them with high-resolution imagery from GEE, ensuring the accuracy of LU/LC classes, such as water bodies, high vegetation, and low vegetation. The time series GEE reference map enabled a detailed review and validation of the threshold values by enhancing the consistency of the results through comprehensive analysis. This comparative process involved the ALST-W classified areas with visually assessed reference points in GEE to enhance thresholds for precise LU/LC representation. Tables 8 and 9, and 10 display the classified samples of LU/LC classes using the proposed ALST-W, LST, and MNDWI indices for 2020, 2022, and 2024 concerning the GEE reference map analysis. Figure 12 presents the reference map highlighting the few randomly selected sampling points in GEE. This visualization improves understanding of the GEE reference map for each LU/LC class placeholder in the research region. The accuracy of the predicted ALST-W map was assessed by cross-verifying it with the reference map generated using GEE. A pixel-wise confusion matrix was computed to assess the classification performance. The overall accuracy achieved was 95.04% for 2020, 95.01% for 2022, and 95.15% for 2024, resulting in an average accuracy of 95.06% and an average misclassification rate of 4.94%. The corresponding confusion matrices for 2020, 2022, and 2024 are shown in Fig. 13. Classification accuracy and misclassification values across years are summarized in Table 11. The proposed ALST-W map provided good classification accuracy by validating its efficiency in capturing spatial thermal patterns. The results confirm that the ALST-W index reliably classifies and distinguishes between the water bodies and vegetative areas, making it a valuable index for temperature-based LU/LC analysis.

**Table 7:** Distribution characteristics of land cover classes based on ALST-W thermal histogram analysis (2020–2024).

Year	Water bodies (ALST-W < 28 °C)	High vegetation (28 °C ≤ ALST-W < 34 °C)	Low vegetation (ALST-W ≥ 34 °C)
2020	Minimal presence, nearly flat curve near 28 °C	Limited coverage, low to moderate frequency	Dominant class with a strong peak between 36–38 °C
2022	Noticeable but low-frequency spread below 28 °C	Dominant class with a peak around 33–34 °C	Moderately present; begins just above 34 °C
2024	Clear presence with wider spread down to 24 °C	Significant, but narrower than in 2022	More frequent than in 2022, peak slightly above 35 °C

**Table 8:** Classified samples of LU/LC features using ALST-W, LST, and MNDWI indices for the year 2020.

Latitude	Longitude	ALST_W_Value	LST_Value	MNDWI_Value	Class_Label	Class_Name
12.59137429	78.80902882	27.619265	28.903528	0.02842634	1	Water bodies
12.5911016	78.80820765	27.871507	29.903135	0.0631628	1	Water bodies
12.5908289	78.80930255	27.737047	29.825018	0.108797126	1	Water bodies
12.5851023	78.8120398	27.994377	29.441422	0.044704504	1	Water bodies
12.58482961	78.81313471	27.224103	29.365606	0.034150403	1	Water bodies
12.58455691	78.81176608	27.862467	29.424568	0.05621007	1	Water bodies
12.58455691	78.8120398	27.36369	28.624382	0.026069293	1	Water bodies
12.58374	78.8120398	27.891731	29.004015	0.011228385	1	Water bodies
12.58347	78.81176608	27.90314	29.493786	0.059064608	1	Water bodies
12.58319	78.81176608	27.889618	29.50547	0.06158533	1	Water bodies
12.60446365	78.82709471	33.48283	32.67821	– 0.18046205	2	High vegetation
12.60419096	78.81532451	33.16568	32.52388	– 0.26418018	2	High vegetation
12.60419096	78.82627353	32.818455	31.83314	– 0.09853143	2	High vegetation
12.60419096	78.82654725	32.339577	31.64907	– 0.06905075	2	High vegetation
12.60419096	78.82682098	33.58803	32.233574	– 0.13544586	2	High vegetation
12.60337287	78.81559824	31.484562	30.451588	– 0.10329736	2	High vegetation
12.60310018	78.81559824	31.430689	30.135668	– 0.12950213	2	High vegetation
12.60310018	78.81587196	32.149548	30.73565	– 0.14138982	2	High vegetation
12.60228209	78.81450333	33.731037	31.642855	– 0.20881829	2	High vegetation
12.60228209	78.81477706	32.814056	31.057896	– 0.17561609	2	High vegetation
12.60446365	78.81860922	38.092113	35.049133	– 0.30429795	3	Low vegetation
12.60446365	78.81888294	38.381317	35.17637	– 0.32049483	3	Low vegetation
12.56628635	78.81121863	37.601772	34.476856	– 0.31249157	3	Low vegetation
12.56628635	78.81149235	37.281113	34.37162	– 0.30109492	3	Low vegetation
12.56710443	78.80656529	37.371918	34.11127	– 0.32606477	3	Low vegetation
12.56710443	78.80574412	37.24743	34.08363	– 0.31637973	3	Low vegetation
12.56710443	78.80711275	37.287018	34.035965	– 0.32510546	3	Low vegetation
12.56737713	78.80574412	37.24743	34.08363	– 0.31637973	3	Low vegetation
12.56737713	78.80601784	37.10467	34.104088	– 0.30005834	3	Low vegetation
12.56764982	78.81258725	37.895466	34.819096	– 0.3076371	3	Low vegetation

**Table 9:** Classified samples of LU/LC features using ALST-W, LST, and MNDWI indices for the year 2022.

Latitude	Longitude	ALST_W_Value	LST_Value	MNDWI_Value	Class_Label	Class_Name
12.59137	78.809029	27.368717	28.116741	0.07480249	1	Water bodies
12.5911	78.809029	26.368717	28.116741	0.07480249	1	Water bodies
12.58483	78.811766	27.9922	28.284185	0.1291985	1	Water bodies
12.58374	78.811492	27.88308	28.41148	0.15284002	1	Water bodies
12.58292	78.811766	26.339941	28.83379	0.14938489	1	Water bodies
12.58401152	78.81176608	27.21981	28.70889	0.148908	1	Water bodies
12.58401152	78.8120398	26.78987	28.270971	0.1481102	1	Water bodies
12.58373883	78.81149235	27.88308	28.41148	0.15284002	1	Water bodies
12.60064592	78.80820765	27.138939	28.913412	0.07744741	1	Water bodies
12.59164699	78.8087551	27.55254	28.682035	0.112949595	1	Water bodies
12.60446	78.813956	33.547337	31.356176	– 0.21911618	2	High vegetation
12.60419	78.82162	33.21868	31.272974	– 0.19457087	2	High vegetation
12.6001	78.828463	33.385883	31.895409	– 0.24904758	2	High vegetation
12.59001	78.822715	33.74549	32.208527	– 0.25369647	2	High vegetation
12.58974	78.803007	33.484795	31.993376	– 0.24914187	2	High vegetation
12.57992	78.803281	33.309322	31.043829	– 0.2265494	2	High vegetation
12.57992	78.807386	33.007477	32.04445	– 0.29630277	2	High vegetation
12.57365	78.806018	33.296333	32.80692	– 0.24894142	2	High vegetation
12.5701	78.814777	33.000957	31.58131	– 0.24196455	2	High vegetation
12.56983	78.803281	33.092163	30.17382	– 0.19183406	2	High vegetation
12.58701117	78.81779	35.96914	33.87334	– 0.30957994	3	Low vegetation
12.58701117	78.81806	36.340275	34.155193	– 0.3185081	3	Low vegetation
12.5741945	78.81122	35.675026	34.22522	– 0.3449808	3	Low vegetation
12.5687406	78.8093	34.269814	33.136227	– 0.31335887	3	Low vegetation
12.56983138	78.81396	34.148792	33.937595	– 0.32111973	3	Low vegetation
12.56983138	78.80958	34.76393	31.71969	– 0.30442426	3	Low vegetation
12.58701117	78.81751	35.56951	33.59929	– 0.3070224	3	Low vegetation
12.58701117	78.81779	35.96914	33.87334	– 0.30957994	3	Low vegetation
12.58701117	78.81806	36.340275	34.155193	– 0.3185081	3	Low vegetation
12.58701117	78.81834	36.340275	34.155193	– 0.3185081	3	Low vegetation

**Table 10:** Classified samples of LU/LC features using ALST-W, LST, and MNDWI indices for the year 2024.

Latitude	Longitude	ALST_W_Value	LST_Value	MNDWI_Value	Class_Label	Class_Name
12.59164699	78.80820765	27.133322	27.843805	0.07104843	1	Water bodies
12.59137429	78.80820765	27.133814	27.844297	0.07104843	1	Water bodies
12.58292074	78.81176608	26.30008	27.711843	0.14117648	1	Water bodies
12.56628635	78.80547039	27.13827	27.983253	0.08449848	1	Water bodies
12.5851023	78.81313471	25.522402	27.549412	0.20270102	1	Water bodies
12.58482961	78.81176608	25.987654	27.133583	0.11459293	1	Water bodies
12.5911016	78.80902882	26.419453	27.105808	0.06863549	1	Water bodies
12.5908289	78.80793392	26.249718	27.57804	0.13283208	1	Water bodies
12.58373883	78.81149235	26.481323	27.98252	0.15011954	1	Water bodies
12.59137429	78.80902882	26.418406	27.104761	0.06863549	1	Water bodies
12.60446365	78.81395588	33.260918	33.585255	– 0.26756626	2	High vegetation
12.56628635	78.80985	32.375874	31.831732	– 0.25441426	2	High vegetation
12.56628635	78.81012373	33.06658	30.212786	– 0.28537968	2	High vegetation
12.57010408	78.81477706	33.664776	31.073204	– 0.2591573	2	High vegetation
12.56983138	78.80328059	31.916935	30.336277	– 0.25806576	2	High vegetation
12.58019379	78.81833549	32.09588	30.677341	– 0.24185376	2	High vegetation
12.5799211	78.80328059	32.35709	30.372131	– 0.19849572	2	High vegetation
12.59001082	78.8227151	33.542606	31.781435	– 0.27611706	2	High vegetation
12.58973812	78.80300686	32.708332	30.153795	– 0.25545374	2	High vegetation
12.60010053	78.82846333	33.220165	31.269737	– 0.29504278	2	High vegetation
12.60419096	78.8216202	36.744415	34.081223	– 0.31631913	3	Low vegetation
12.60391826	78.80902882	37.314022	34.086044	– 0.32279792	3	Low vegetation
12.57392181	78.81286098	37.251705	34.05042	– 0.32012847	3	Low vegetation
12.57364911	78.80601784	36.850628	34.086025	– 0.36646014	3	Low vegetation
12.57692145	78.80793392	37.70133	34.01266	– 0.36886656	3	Low vegetation
12.57473989	78.80601784	37.210445	34.02689	– 0.3183557	3	Low vegetation
12.58019379	78.81067118	37.20194	34.01001	– 0.31919292	3	Low vegetation
12.5799211	78.80738647	37.300797	34.118965	– 0.31818333	3	Low vegetation
12.60064592	78.82545235	38.06657	35.12556	– 0.30410103	3	Low vegetation
12.59219238	78.80574412	38.08389	34.643406	– 0.34404835	3	Low vegetation

**Table 11:** Temporal evaluation of classification accuracy and errors using Pixel-based metrics.

Year	Total pixels	Correctly classified	Overall accuracy (%)	Misclassification rate
2020	9138	8685	95.04	4.96
2022	9138	8682	95.01	4.99
2024	9138	8695	95.15	4.85
Average accuracy			**95.06**	**4.94**

## Discussion

This research introduces an advanced approach by unifying temperature data from water bodies and high and low vegetation through the ALST-W index. The results from the existing LST maps typically focus on temperature variations associated with high and low vegetation, often neglecting the unique thermal properties of water bodies. The ALST-W system resolves this by distinctly isolating water body temperatures from the broader dataset, delivering a more refined and precise surface temperature profile for our research region. By integrating the LST with a scaled MNDWI, the ALST-W index enhances the understanding of temperature mapping to water-related effects. This integration aids in differentiating between the hot, dry areas and cooler, moisture-rich zones by refining the spatial clarification in mixed LU/LC regions. The ALST-W index reveals the clear temperature differences, with the highest readings observed in low vegetation areas, moderate values in high vegetated areas, and significantly lower temperatures in water bodies, especially in non-forested zones. This refined level of detail proves essential for applications related to ecosystem monitoring, climate studies, and the management of water resources, where accurate representation of water body temperatures plays a critical role. Table 12 shows a comparison between LST and ALST-W in spatial data analysis. It points out that ALST-W takes a broader approach by including water body temperatures and high and low vegetation, while LST has a narrower focus. In addition, the ALST-W index serves as a spatial driver map within predictive frameworks, facilitating the representation of temperature distributions across various land types in a single raster map. Unlike traditional LST maps, which offer a narrower perspective, ALST-W provides a more comprehensive and integrative temperature dataset, enhancing the analysis of spatial and temporal temperature patterns.

**Table 12:** Comparison of LST and ALST-W in spatial data analysis.

Comparison aspect	LST^55^	ALST-W [Ours]
Focus	High and low Vegetation soil temperatures.	Comprehensive analysis of high and low vegetation, and water bodies.
Water body temperature	Not distinctly represented.	Clearly defined and separately analyzed.
Data integration	Limited to specific land cover types.	Unified dataset across all land types.
Predictive capability	Minimal support for predictive modeling.	Enhances predictive frameworks for accurate temperature forecasting.

The ALST-W framework improves the accuracy of the LST calculations by incorporating the distinct thermal characteristics of various LU/LC types, mainly water bodies. This approach reduces errors, improves spatial resolution, and captures the surface temperature variability more efficiently by establishing the ALST-W as a robust tool for research and practical applications. It improves the identification of confined heat differences and supports informed strategies in managing land and water resources. The proposed ALST-W index offers high adaptability by making it suitable for application across various landscapes, including forests, coastal regions, and highly urbanized areas. The methodological flexibility of the ALST-W index supports the accurate classification and monitoring across different biological and climatic backgrounds by enhancing its utility for broad environmental analysis.

Along with the vegetation and water bodies, key impact variables such as soil moisture, urban index, elevation, slope, wind speed, air temperature, humidity, rainfall, and population data significantly influence ALST-W dynamics. These impact factors contribute to the various temperature patterns across different LU/LC types and timeframes by determining the ecological and climatic conditions. Integrating these variables into the ALST-W framework can significantly improve the precision and relevance of surface temperature analysis. In future work, we aim to integrate these impact elements to build a more robust and scalable temperature monitoring model. This development will enhance its applications in climate modeling, land resource management, and sustainable land-use planning. The ALST-W index performs fine but faces challenges in the complex LU/LC types like urban and built-up areas, where MNDWI has restricted influence on thermal patterns. Our future research will improve the index for varied landscapes and extend validation across seasons and regions. Also, our upcoming research will focus on including the multi-temporal datasets for several seasons and years. This will permit the improved assessment of ALST-W’s temporal steadiness and ability to analyze the long-term surface changes.

## Conclusion

This research aimed to evaluate the influence of water bodies on the LST map using the ALST-W index. We developed an effective approach to isolate water body temperatures from the LST map. Using Landsat data from the non-forest-covered region of Javadi Hills, we analyzed ALST-W maps for 2020, 2022, and 2024. A thresholding mechanism differentiates the temperature values precisely to water bodies and high and low vegetated areas. Histogram analysis identifies the dominant thermal signatures and their distribution across 2020, 2022, and 2024. The reference map analysis validates the spatial accuracy by ensuring a robust classification of LU/LC features. The validation against the ALST-W maps through the GEE reference data yielded a good accuracy of 95.06% with a misclassification error of 4.94%. The findings on changes in water bodies and high and low vegetation can assist government officials in land resource management and environmental protection. Future research will focus on extracting the predicted temperature values from urban and built-up areas through different impact variables in conjunction with water bodies and high and low vegetation for different multi-temporal datasets.

## Data Availability

The Landsat 9/8 datasets which are used in this research work is openly available in USGS, United States of America (https://earthexplorer.usgs.gov). The spatial boundary data (shapefiles) were obtained from DIVA GIS, a free and open-access spatial data source (https://www.diva-gis.org). Reference data for accuracy assessment and sample-based analysis were accessed and processed using Google Earth Engine (https://earthengine.google.com), a cloud-based geospatial analysis platform.
